# Dose REduction strategy of subcutaneous TNF inhibitors in rheumatoid arthritis: design of a pragmatic randomised non inferiority trial, the DRESS study

**DOI:** 10.1186/1471-2474-14-299

**Published:** 2013-10-24

**Authors:** Alfons A den Broeder, Noortje van Herwaarden, Aatke van der Maas, Frank HJ van den Hoogen, Johannes W Bijlsma, Ronald F van Vollenhoven, Bart JF van den Bemt

**Affiliations:** 1 Department of Rheumatology, Sint Maartenskliniek, PO box 9011, Nijmegen 6500 GM, The Netherlands; 2 University Medical Center Utrecht, Utrecht, the Netherlands; 3 The Karolinska Institute, Stockholm, Sweden; 4 Department of Pharmacy, Sint Maartenskliniek, Nijmegen, The Netherlands

**Keywords:** Rheumatoid arthritis, Dose reduction, Discontinuation, Anti TNF, Spacing, Randomised controlled trial, Non-inferiority, Cost minimalisation, Design, Decremental cost effectiveness ratio (DCER)

## Abstract

**Background:**

Preliminary, mostly uncontrolled studies suggest that dose reduction or discontinuation of tumour necrosis factor blockers can be achieved in a relevant proportion of patients with RA without loss of disease control. However, long term safety, cost effectiveness and feasibility in clinical practice remain uncertain.

**Methods/Design:**

This study is a 18-months pragmatic, non-inferiority, cost minimalisation, randomized controlled trial on dose reduction and discontinuation of the subcutaneous tumour necrosis factor (TNF) blockers adalimumab and etanercept in RA patients with low disease activity. 180 RA patients with low disease activity (DAS28 < 3.2 or clinical judgment of the rheumatologist) are randomized 2:1 to either increased spacing and eventually discontinuation after 6 months of the TNF blocker, and usual care. Implementation is done in routine daily care, using treat to target and feedback implementation in both treatment arms. The primary outcome is non-inferiority (NI margin 20%) in cumulative incidence of persistent (> 3 months) RA flare, according to a recently validated DAS28 based flare criterion (DAS28 change > 1.2, or DAS28 increase of 0.6 and current DAS28 ≥ 3.2). Secondary outcomes include mean disease activity, function, radiographic progression, safety and cost effectiveness. Cost per quality adjusted life year (QALY) differences between groups are expressed as a decremental cost effectiveness ratio (DCER), i.e. saved costs divided by (possible) loss in QALY.

**Discussion:**

The design of this study targeted several clinical and methodological issues on TNF blocker dose de-escalation, including how to taper the TNF blockers, the satisfactory control condition, how to define flare, implementation in clinical practice, and the choice of the non-inferiority margin. Pragmatic cost minimalisation studies using non-inferiority designs and DCERs will become more mainstream as cost effectiveness in healthcare gains importance.

**Trial registration:**

Dutch Trial Register NTR3216, The study has received ethical review board approval (number NL37704.091.11)

## Background

Tumour necrosis factor blocking agents (TNF-blockers) have proven to be effective and safe pharmacological interventions in the treatment of rheumatoid arthritis (RA). As these agents improve clinical, functional and radiographic outcome, TNF-blockers have become an integral part of the standard of care of RA. However, TNF-blockers are also associated with (sometimes dose dependant) adverse effects including injection site reactions, increased risk of infections and non melanoma skin cancer/lymphomas, rare severe adverse events and high costs [1-3]. Optimal use of these drugs is therefore warranted, including the right dose for the right patient [4]. Elective dose reduction in the context of low disease activity is however up to recently very uncommon in daily clinical practice [5].

Emerging data, mostly uncontrolled, has indicated that dose reduction or discontinuation of TNF blockers [6-20] can be achieved in a relevant proportion of patients with RA without loss of disease control. This seems similar between the three most used anti-TNF agents infliximab, adalimumab and etanercept (no data are available on certolizumab and golimumab), although the proportion of patients in whom the drug can be safely tapered seems to depend on the design of the study and context (especially authorized or higher than authorized dosage, dose reduction or stopping, and in early or established RA).

The fact that dose reduction or discontinuation can be successful could be expected for several reasons [4]. In clinical phase II/III studies, lower than registered anti-TNF dosages have been shown to result in good response in sizable proportions of patients [21-23]. So, maintenance of clinical efficacy on lower dosages is to be expected in many patients. In addition, patients sometimes improve independently of the installed treatment, as witnessed by the improvement that is found in placebo arms of clinical trials [21-23]. This improvement is in part spontaneous improvement (regression to the mean) or due to concomitant DMARD or glucocorticoid therapy, but also caused by the placebo effect (expectation bias) [24].

Although data on dose reduction is increasing, a number of aspects of dose tapering strategies in TNF blockers are still not well known thus far. Is reinstallment of the TNF blocker safe and effective? Is reducing the dose while maintaining clinical response associated with more radiographic joint damage in the long-term? Can these strategies be implemented in daily clinical care, and what is the cost effectiveness compared to usual care? To answer these questions, we designed a pragmatic RCT, the results of which will be presented in a separate paper. The primary aim of this study is to demonstrate non-inferiority of a dose reduction strategy compared to usual care with regard to persistent disease flare.

During the design of this RCT, a number of issues had to be addressed and in this paper we would like to describe in detail the study design, and motivate and discuss some of the design choices that were made.

## Methods/Design

This pragmatic, open, randomised, controlled, stratified non-inferiority strategy trial with cost effectiveness analysis is currently being performed (inclusion finished October 2012) at the departments of rheumatology of the Sint Maartenskliniek in the cities of Nijmegen and Woerden, the Netherlands. The study has received ethical review board approval (number NL37704.091.11) and has been registered (Dutch Trial Register NTR3216). A data safety monitoring board (DSMB) is installed. Every three months data on recruitment, efficacy, safety, protocol adherence and protocol updates and all aspects concerning GCP are reviewed with three independent DSMB members, an internal medicin physician, a pharmacist and an epidemiologist.

**Figure 1:**
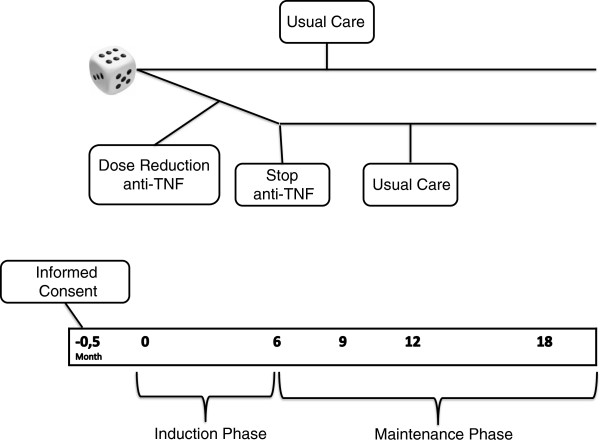
Design of the induction and maintenance phase.

We made a distinction in an induction phase (months 0-9 after the first dose reduction) and a maintenance phase (months 6-18), because the cost effectiveness is very different between these two time periods [14] (Figure 1). In the induction phase, medication costs are still high, patients are sometimes seen more often, and quality of life might be compromised by temporary flares. Therefore, the cost effectiveness ratio found in the stable maintenance phase can be better interpreted for subsequent years.

### Objectives

The aim of the study is to test whether a tight control strategy with protocollised dose reduction and discontinuation in RA patients using TNF blockers is non-inferior with regard to disease control compared to a strategy without dose reduction attempt, and superior with regard to cost effectiveness. This translates in the following research questions:

#### ***Primary objective***

– To assess whether the difference in cumulative incidence in persistent RA flares with a duration > 3 months between the intervention group and the usual care group of RA patients does not exceed the non-inferiority margin of 20% after 18 months of follow up.

#### ***Secondary objectives***

– To compare the cumulative incidence of patients fulfilling flare criteria in the intervention and the usual care group after 9 months and after 18 months of follow up.

– lTo compare the cumulative incidence of patients with persistent RA flare resulting in change of biological between the intervention and the usual care group after 9 months and after 18 months of follow up.

– To compare the proportion of patients with a DAS28 < 3.2, DAS28 < 2.6 and fulfilling remission criteria according to ACR/EULAR criteria between the intervention and usual care group at 9 and 18 months follow up.

– To compare the mean DAS28 and the mean time averaged DAS28 between the intervention and usual care group at 9 and 18 months follow up.

– To compare the proportion of patients using NSAIDs, glucocorticoids or DMARDs between the intervention and usual care group at 9 and 18 months follow up.

– To compare the mean Health assessment questionnaire disability index (HAQ-DI) between intervention and usual care group at 9 and 18 months follow-up.

– To compare the proportion of patients with a change in modified Sharp-van der Heijde Score exceeding the minimal clinical important change (MCIC) between the intervention and usual care group at 18 months.

– To compare the proportion of patients developing adverse events with special attention for allergic/injection reactions between the intervention and usual care group.

– To estimate the decremental cost effectiveness ratio of a protocollised dose reduction/withdrawal strategy of adalimumab or etanercept compared to usual care for the 9 months induction phase and for the 12 months maintenance phase.

#### ***Study design rationale***

The comparison made in this study is between usual care and an alternative strategy (dose reduction) that aims to preserve, but not improve, disease control, whilst minimising the amount of TNF blocker. Therefore, a non-inferiority design (“can we achieve the same effect with less effort”) instead of the classic superiority RCT design (“can we do better with more effort”) was chosen. Of note, this is an adaptation of the original registered research protocol.

#### ***Study outcome rationale***

As disease control is the domain that should remain non-inferior, an outcome measure of disease control had to be selected as primary outcome. Several options come to mind, each with specific advantages and drawbacks.

A straightforward option would be a comparison of disease activity at study end, like for example mean DAS28 with non-inferiority margin of 0.3 DAS28 points. However, it can be expected that at study end no difference is found between the two strategies because patients are treat-to-target, but that flaring is much more frequent during the study in the dose reduction arm. Comparison of time integrated DAS28 might be therefore a better alternative, with the bonus of lower sample size requirements due to the repeated measurements [18]. However, disease activity AUC values are harder to interpret and less informative for a practising clinician than percentage of patients with a flare. The latter also facilitates the calculation of numbers needed to treat/harm (NNT/NNH), thus making results easier to interpret and communicate to patients and physicians alike.

Another option would therefore be to use cumulative incidence of flares as primary outcome, and this is chosen in the majority of dose reduction and discontinuation studies. However, temporary flares that improve after reinstallment of therapy and without a large impact on overall disease control will inevitably occur more frequent in a dose reduction arm, but are clinically less relevant than persisting flares. We therefore chose to assess non-inferiority with respect to cumulative incidence of persisting flare, which is defined as a flare according to the DAS28 based OMERACT validated flare criterion persisting for at least 3 months (independent of treatment changes).

An interesting alternative for a primary outcome of non-inferior disease control would be to prove superior cost effectiveness of the alternative strategy. This would require proving that either the alternative strategy does not lead to loss of Quality Adjusted LifeYears (QALY) but saves costs (dominant strategy), or that the ratio of saved costs and loss of QALY is favourable. Of note, instead of the common term incremental cost effectiveness ratio (ICER), this approach results in a decremental cost effectiveness ratio (DCER), that states the amount of costs saved per lost QALY. The use of this kind of analyses as primary research question is however challenging due to several issues, and therefore very infrequently employed [25]. Firstly, due to the bootstrapping methods that are used to calculate the DCER, a formal sample size calculation is not possible. Secondly, quality of life is only moderately correlated with RA disease activity, posing a real chance of false reinsurance when quality of life does not seem to be compromised. Thirdly, although ICERs of under 40.000 euro per gained QALY are widely accepted as cost-effective , no such clear-cut threshold is available for DCERs. Although the symmetry of using also 40.000 as threshold for DCER seems attractive, as these saved cost can be used elsewhere in health care system to get more quality of life improvement than was lost, it seems awkward to provide suboptimal therapy in relatively low cost effectiveness ranges. However, it can be argued that when the situation was reversed, e.g., when usual care would be to attempt dose reduction, the alternative of continuing medication would not be considered cost-effective when data shows an ICER clearly over 40.000. Interpretation of DCER seems therefore coloured by anchoring to either the existing and/or the alternative strategy. Anyway, a high DCER of for example > 100.000 saved per lost QALY would probably meet wide acceptance from a cost effectiveness point of view [25].

### Patients, eligibility and generalisability

All patients are eligible to enter the study if they are diagnosed with rheumatoid arthritis (either 2010 ACR RA and/or 1987 RA criteria and/or clinical diagnosis of the treating rheumatologist, fulfilled at any time point between start of the disease and inclusion), and of any disease duration, use etanercept or adalimumab in any stable dose for at least 6 months, and are in low disease activity state [26,27]. Previous dose reduction of the TNF blocker is allowed when more than 6 months ago. All background medication including DMARDs (either monotherapy or in combination) and prednisone equivalent up to 5 mg are allowed during the study and have to be stable for at least four weeks before inclusion. Consenting patients are randomly allocated in a ratio of 2:1 to the intervention or the control group, stratified for TNF blocker (adalimumab and etanercept).

We have chosen to operationalise low disease activity state by either a DAS28CRP < 3.2 or judgment of low disease activity by the rheumatologist at two subsequent visits at least 3 month apart. This means also patients with a DAS28 ≥ 3.2, but judged to have a low disease activity by their treating rheumatologist, are eligible for inclusion. This is firstly because several biologic registries show that mean DAS28 in TNF Blocker treated patients in clinical practice is around 3.2 [28]. This means that over 50% of the patients has a DAS28 ≥ 3.2, and even more are not in remission, but that both the patient and rheumatologist judge the disease activity to be low enough not to warrant switch of treatment. Limiting inclusion to only patients with DAS28 < 3.2 or even < 2.6 would therefore limit generalisability to current treatment practices. It might be rebutted that these higher disease activity scores reflect undertreatment and thus result in suboptimalisation. A DAS28 can however be inflated by OA associated joint pain, concomitant fibromyalgia or spuriously elevated ESR. This is also the rationale for use of DAS28CRP instead of DAS28 ESR. Another important consideration is that we treat RA patients for two reasons: firstly to improve current signs and symptoms, and secondly to prevent joint damage. With respect to the first reason, it is important to note that the patient acceptable symptom state of the DAS28, has been found to be around 3.5 to 3.9, clearly higher than the currently advocated treatment aims of 2.6 or 3.2 [29]. For prevention of joint damage it is important to realize that not all patients have high risk of erosive disease and a need for prognosis modification [30]. Therefore, accepting somewhat higher disease activity can be legitimate and is indeed part of clinical practice. Of note, per October 19, 2012, all patients (n = 180) have been enrolled and randomised. Mean DAS28CRP is 2.2, and 8% of patients had a DAS28 > 3.2.

Because we aimed for maximal generalisability, we chose to only exclude patients if they have co morbidity which also requires treatment with anti-TNF and thus prevents dose reduction (e.g. Crohns disease), or when it is to be expected that the outcome cannot be measured (short life expectancy, planned major surgery).

### Interventions

#### ***Control***

Patients in the usual care group will continue treatment by their own rheumatologist, following a standardized protocol based on the tight control/treat-to-target principle and aiming to maintain low disease activity. Visits are planned every 3 months and patients are encouraged to contact the outpatient clinic if they experience more complaints in between visits. CRP based DAS28 measurements are provided on the day of the outpatient visit to the treating rheumatologists. The standardized protocol offers protocollised treatment suggestions when there is a flare, including a treatment algorithm and dosing of the drugs. The research physician monitors protocol adherence of the rheumatologists and will - where needed - give feedback and advice to the rheumatologist. Treatment choices, however, are left to the discretion of the treating rheumatologist. This way it is ensured that positive study results can be achieved also in clinical practice. Treatment will be changed in case of a confirmed flare (the definition of flare will be discussed separately). A one time flare will be, if necessary, bridged with glucocorticoids. Also, dose reduction or stopping, for any reason, was allowed in the control arm, as this is sometimes-although infrequent-also part of usual care (5). In summary, the control condition exist of tight control by patients own rheumatologist, and all treatment choices are allowed.

Of note, although called usual care, tight control care has thus far not consistently been implemented in clinical care in the Netherlands [31]. However, opting for a standard care arm without tight control would result in underestimation of possible drawbacks of dose reduction, as these effects would be ameliorated by the difference in tight control.

#### ***Intervention***

The intervention group receives identical care as the control group, with addition of a dose reduction and withdrawal strategy protocol and feedback and advice to treating rheumatologist. This strategy is directly adapted from the Dutch Society of Rheumatology biological guideline [32]. If a patient uses adalimumab, the interval will be stepwise increased every three months from 14 to 21 to 28 days, after that the adalimumab will be stopped. For etanercept, the interval will be increased from 7 to 10 to 14 days and stopped thereafter. If a patient uses a different dose regimen at start, an alternative dose reduction strategy is used. Patients already on a longer dosing interval will step in at the nearest dosing interval. Patients on a shorter dosing interval will also stepwise increase the interval to stop after 6 months using an accelerated strategy. The three month interval is based on data that shows that most of the flaring after dose reduction occurs within 3 months [14].

The dose reduction steps from 100% to 66% and 50% and thereafter stop are based on the notion that relatively small dose reduction seems to be feasible in a large proportion of patients, and therefore will lead to sizable reduction in total volume and costs of the TNF blocker [14,20]. Indeed, although stopping the TNF blocker obviously saves more drug per patient than dose reduction, the total volume of saved drugs is the same in patients that stop or are only dose reduced, because the latter group is much larger [14]. Also, although we cannot substantiate this, dose reduction until stop feels more appropriate for patients than just stopping the drug.

When a confirmed flare occurs, the interval is decreased back to the last effective interval. When there is still no improvement of disease activity eventually after reintroduction of the original interval and dose, the patient will be advised to switch to the next biologic or DMARD according to the treatment protocol.

### Outcome measures

The outcome measures used in this study include disease activity (DAS28CRP based RA Flare criterion, mean and time integrated DAS28CRP), function (HAQ DI), radiological damage (Modified Sharp van der Heijde score, MSvdH), Adverse events (CTC 4.0 criteria), utility (Euroqol 5D 5 L) and costs [33-39].

#### ***Definition of flare***

RA Flare in this study is defined in both treatment arms as a DAS28 increase compared to baseline of more than 1.2 or a DAS28 increase of more than 0.6 with a current DAS28 ≥ 3.2 at two separate timepoints at least 4 weeks apart. This criterion has been validated recently, and has been shown to have the optimal tradeoff between sensitivity and specificity, and the best construct and criterion validity [40]. As it has been shown that flares are frequently temporary and occur and disappear without regimen change, a flare is only considered a flare if it is confirmed after at least 4 weeks [11]. Patients are however not left untreated, and when a flare in disease activity occurs, all bridging therapy including i.a. or im steroids or NSAID can be given and are allowed. Of note, flares are defined with change compared to baseline, not compared to the last visit, to prevent undertreatment in patients with a slight increase in disease in subsequent visits.

When the flare is confirmed after 4 weeks, in both treatment arms patients receive optimal treatment for the flare, for example protocollised reinstallment or dose increase of the TNF blocker or change to another biologic or DMARD. Only patients in whom the flare persist longer than three months (in spite of all treatment intensivation) are classified as having persistent flare, the primary outcome of this study.

#### ***Secondary outcomes***

##### 

**Cost effectiveness** A separate calculation will be made for the 9 months induction phase and the 12 months maintenance phase for the reasons described above. The cost analysis includes both direct and indirect costs, from a society perspective, and consist of two main components: determination of volumes of care and determination of cost prices for each volume of consumption. Volumes of care (registered out-patient clinic visits, medication use, and work-related absenteeism) are multiplied with the cost prices to calculate costs. Cost prices for medication are retrieved from the Dutch National tariff list provided by the Dutch Board of Health Insurances. The standard cost prices from the ‘Dutch Guideline for Cost Analyses’ are used for valuation of hospital related care and work-related absenteeism.

##### 

**Radiographic damage** Change in radiographs of hands and feet between baseline and study end are compared between intervention and usual care by calculating the change in MSvdH score, scored chronologically in random order [35]. The smallest detectable difference (SDD) within this 18 months timeframe in this group of patients with established RA is expected to be 8 points, comparable with the widely validated minimal clinical important change (MCIC) of 5 point per year [38,39,41]. Proportions of patients showing radiographic joint damage progression exceeding the MCIC are then calculated.

### Assessments

Regular visits are planned at baseline and every 3 months thereafter up to month 18. When a flare occurs, patients are seen within two days, but at least within a week, and additionally an extra visit is planned after 4 weeks. In Additional file 1: Table S1 we outlined all the visits and assessments.

### Randomisation, allocation concealment and blinding

Allocation is stratified for adalimumab and etanercept using stratified block randomization in random sized blocks and a ratio of 2:1. Patients will be randomised by the research physician using a computer-generated randomisation list, which has been transferred to paper sheets and put in sealed envelopes.

This study uses a controlled and randomised, but only partly blinded design, as patients and physicians are not blinded for allocation. Blinding of the assessor performing the joint scores was strived for, and this was done by instructing both patients and the independent joint assessor to first score the DAS28CRP, before assessing medication changes and adverse effects.

Although triple blinding (patients, physicians, researcher) would methodologically be preferable, this is unfeasible because dose reduction in this study is done via increasing the interval instead of decreasing the dosage per injection. The latter would not be sensible, since TNF blockers are given using prefilled injection pens. Blinding of patient and physician is possible when using interval widening, but would be very difficult. The unblinded nature of the study could result in information and attribution bias, as flares in patients in whom the dose is reduced would possible be reported sooner because they would be attributed to the intervention. This bias can however fortunately only lead to overestimation of the drawbacks of a dose reduction strategy, not underestimation, and therefore the higher risk of bias was accepted.

### Sample size

The null hypothesis in this study is that the intervention is inferior compared to the control arm by more than the non-inferiority margin δ (H0: μ1-μ2 > δ). The alternative hypothesis rejects this null hypothesis (H1: μ1-μ2 ≤ δ), thus proving non-inferiority. The sample size calculation is motivated as follows [42] using the fomula as shown.

$$n_{1}=kn_{2}$$

$$n_{2}=\frac{{\left(Z_{a}+Z_{\beta }\right)}^{2}}{{\left(\epsilon -\delta \right)}^{2}}\left[\frac{p_{1}\left(1-p_{1}\right)}{k}+p_{2}\left(1-p_{2}\right)\right]$$

We estimated that 20% (p_1_ = 0.80) of patients will experience the primary outcome in the usual care arm (persistent flare), with an estimated 15% (p_2_ = 0.85) of patients with this outcome in the intervention arm. Applying one sided testing, an alpha of 0.05 (Z_α_ = 1.64, non-inferiority testing one sided), power 1-beta 0.8 (Z_β_ = 0.84), an inferiority margin of 20% (δ = -0.2), and randomisation ratio of 2:1 intervention versus control (k = 2) resulted in n = 114 and n = 57 for intervention and control arm. Accounting for a 10% drop-out, we choose to include 180 patients in total.

The estimation of proportion of primary outcome in both groups and magnitude of the non-inferiority margin (δ) are motivated as follows. Although clear data are absent, based on the few dose reduction studies that included a control group, and on previously published drug survival curves for adalimumab and etanercept in our population, we estimated that after 18 month in the usual care group 15% of patients would have changed their biologic therapy due to insufficient disease control [12,15,16,28]. We furthermore assumed that 15% of patients in the intervention group can ultimately stop the drug (without persistent flare), resulting in cumulative incidence of 85% flares in the group of patients with partial dose decrease. Of these patients, we estimate that 5% will not respond to reinstallment of the TNF blocker and experience a persistent flare. Thus, we expect a cumulative incidence of persistent flare of 15 + 5 = 20% in the intervention arm.

A difference in persistent flare of over 20% between usual care and dose reduction would constitute a clinical relevant non-inferiority margin in our opinion. The underlying reasoning is, that it is to be expected that half of the patients who start another biologic for a flare will show response again within three months (21–23). Half of 20% of the patients would therefore experience a persistent flare, a more prolonged period with uncontrolled disease activity, resulting in a NNH of 10. In our clinical view, this seems to balance nicely with an expected chance of being able to reduce the dose or stop the drug of approximately 60 and 15% respectively (NNT 1.5 and 6 respectively), as much more patients are expected to benefit than to be harmed using this non-inferiority margin. The ratio of 2:1 for intervention and control sample size is chosen to be able to include more determinants in a prediction model for successful dose reduction.

### Planned data analysis

All statistical analyses are performed using STATA/IC 10.1 for Windows. Analyses will be done on intention to treat (ITT) basis and also per protocol basis, the latter because ITT analyses might underestimate differences between treatment arms in non-inferiority studies, resulting in a false positive study [25]. Number and reasons for exclusion and dropout are reported to ensure internal validity. Missing data on determinants/covariates will be described using descriptive analyses and missing data mechanisms will be studied. Missing values will be imputed using multiple imputation when meeting the assumption of missing (completely) at random, as imputation will increase precision and possibly reduce bias. Logistic regression models with the indicator variable as outcome and the other variables as covariates will be used to check these assumptions. Multiple imputation using chained equations will then be used to estimate missing values. Descriptive statistics will be provided using mean +/-SD, median (p25-p75) or frequencies/percentages depending on the type distribution of the data.

The primary outcome, cumulative incidence of persistent flare is calculated in both groups. The two proportions are then compared using Fisher exact testing. The cost and quality of life are compared between intervention and usual care group for the induction phase and for the maintenance phase separately. Direct costs and indirect cost will be calculated, as will utility based on EUROQOL-5D-5 L. Thereafter, when the intervention is not a clearly dominated or dominant strategy, a decremental cost effectiveness ratio will be estimated using bootstrapping, expressed as saved costs divided by loss in quality of life.

## Discussion

The development of the current study protocol induced a number of clinical and methodological issues that had to be targeted, and that are specific for the context of this research area. These choices include how to taper the TNF blockers, the satisfactory control condition in light of current treatment practices, how to define flare, implementation in clinical practice, and the choice of the non-inferiority margin. This resulted in a clinical study design that differs in many respects from previously published and ongoing studies in this field.

In addition to the methodological issues mentioned above, some medico-legal issues also arose. According to new legislation based on GCP II, the 2001 EU directive and the subsequent Dutch law on medical research (WMO 2006), clinical studies that study patients using medication, even when given according to current registration demands and not initiated or changed for study purposes, are considered interventional medication studies [43,44]. This label results in a large administrative burden (EUDRACT registration, investigators brochure, increases monitoring demands, and responsibility for medication cost), making investigator driven clinical pharmacological research difficult or even nearly impossible to perform. Paradoxically, this results in less safe and (cost) effective care for the very people these laws are meant to protect, the patients. Inspired by the discussion amongst clinical researchers concerning practical implications of this legislation, medical ethical review boards have developed more lenient approaches to these kind of studies [45,46]. Indeed, because our study does not directly specify what medication to give, but provides a comprehensive treatment strategy and feedback as intervention, because the drugs are given for standard indication, and as the intervention is withdrawal of the drug, the study was considered not an interventional medication study.

In conclusion, healthcare in the next decades will not only be driven by improving outcomes, but also by achieving the same results with less effort and less risk for adverse effects. Non-inferiority studies combined with decremental cost effectiveness analyses – although thus far seldom done and challenging [25]–are the preferential design to achieve this. The widespread and long term use of expensive and sometimes toxic biologicals in chronic inflammatory conditions seems an excellent field to start with improving the cost effectiveness of our current high cost healthcare system. Future research in this topic should include expansion to other biologics and other diseases.

## Abbreviations

ACR: America college of rheumatology; CRP: C-reactive protein; CTC: Common toxicity criteria; DAS28: Disease activity score 28; DMARD: Disease modifying anti rheumatic drug; ESR: Erythrocyte sedimentation rate; EUDRACT: European union drug regulating authorities clinical trials; EUROQOL: European quality of life; GCP: Good clinical practice; HAQ DI: Health assessment questionnaire disability index; I/DCER: Incremental/decremental cost effectiveness ratio; ITT: Intention to treat; MCIC: Minimal clinical important change; NNT/NNH: Number needed to treat/harm; OA: Osteoarthritis; RA: Rheumatoid arthritis; SDD: Smallest detectable difference; TNF: Tumour necrosis factor; QALY: Quality adjusted life years.

## Competing interests

There are no other financial or non financial conflicts of interest other than specified below. This manuscript and the clinical study do not receive funding that results in conflicts of interest.

Alfons A den Broeder: Payment for educational lectures for Roche and MSD (all < 10.000 USD).

Noortje van Herwaarden: none.

Aatke van der Maas: none.

Frank HJ van den Hoogen: received reimbursement for advice from BMS, Pfizer and Roche in the past five years (all < 10.000 USD).

Johannes W Bijlsma: consultant and/or speaker to Abbott, Roche, BMS, UCB, Pfizer and Merck (all < 10.000 USD).

Ronald F van Vollenhoven: research support and/or honoraria: Abbott, MSD, Pfizer, Roche, UCB Pharma.

Bart JF van den Bemt: payment for educational lectures for Roche and MSD.

## Authors’ contributions

AdB is rheumatologist and clinical epidemiologist, and principal investigator and initiator of this RCT. He is medical head of the dept of rheumatology of the St Maartenskliniek and has designed and drafted the manuscript. NvH is MD and the PhD student involved in design and execution of the study, and has drafted the protocol and performed CMO admission. AvdM is rheumatologist and clinical epidemiologist and involved as PhD counsel for Noortje van Herwaarden. She also was involved in preparatory clinical work, and aided in design, drafting and statistics. FvdH is rheumatologist and director of the rheumatology center, and involved in trial rationale, drafting, patient inclusion and organisation. JB, professor in rheumatology in Utrecht and RvV, professor in rheumatology in Stockholm are involved as PhD counsel for Noortje van Herwaarden, and are also cooperating in several national and international clinical studies on the subject. They have been involved in design and drafting. Finally, BvdB is pharmacist and has done preparatory work in this field, is involved as PhD counsel for Noortje van Herwaarden and is also involved in dose optimalisation of TNF blockers and the use of therapeutic drug monitoring as prediction for de-escalation. All authors read and approved the final manuscript.

## Pre-publication history

The pre-publication history for this paper can be accessed here:

http://www.biomedcentral.com/1471-2474/14/299/prepub

## Supplementary Material

Additional file 1: Table S1 Study visits and assessments. Click here for file
