# Supplementary material for: Dose REduction strategy of subcutaneous TNF inhibitors in rheumatoid arthritis: design of a pragmatic randomised non inferiority trial, the DRESS study
Source: BMC Musculoskelet Disord. 2013 Oct 24;14:299. doi: 10.1186/1471-2474-14-299 (PMC4016115; doi:10.1186/1471-2474-14-299)
Supplement: Additional file 1: Table S1 — Study visits and assessments. [file 1471-2474-14-299-S1.doc]

**Additional file 1**

**Table S1** Study visits and assessments

|  | **Month** |  |  |  |  |  |  |  |  |
| --- | --- | --- | --- | --- | --- | --- | --- | --- | --- |
| **Assessment** | -0.5 | 0 | 3 | 6 | 9 | 12 | 15 | 18 | Extra visit  (in case of self reported flare or DAS28 flare at last regular visit) |
| patient information/ informed consent |  |  |  |  |  |  |  |  |  |
| Baseline data |  |  |  |  |  |  |  |  |  |
| Medication |  |  |  |  |  |  |  |  |  |
| DAS28 |  |  |  |  |  |  |  |  |  |
| Flare criteria |  |  |  |  |  |  |  |  |  |
| VAS pain |  |  |  |  |  |  |  |  |  |
| VAS disease activity |  |  |  |  |  |  |  |  |  |
| Judgement disease activity physician and patient in comparison to previous visit |  |  |  |  |  |  |  |  |  |
| HAQ DI Dutch version |  |  |  |  |  |  |  |  |  |
| EUROQOL-5D-3L |  |  |  |  |  |  |  |  |  |
| Health related absence |  |  |  |  |  |  |  |  |  |
| Adverse events |  |  |  |  |  |  |  |  |  |
| Radiographic hands/feet |  |  |  |  |  |  |  |  |  |
| Serum sample |  |  |  |  |  |  |  |  |  |
